# Avian Hepatitis E Virus in Chickens, Taiwan, 2013

**DOI:** 10.3201/eid2001.131224

**Published:** 2014-01

**Authors:** Ingrid W.-Y. Hsu, Hsiang-Jung Tsai

**Affiliations:** National Taiwan University, Taipei, Taiwan (I.W.-Y. Hsu, H.-J. Tsai); and Council of Agriculture, New Taipei City, Taiwan (H.-J. Tsai)

**Keywords:** avian hepatitis E virus, avian HEV, ORF1, ORF2, ORF3, ELISA, seropositive rate, phylogeny, complete genome, viruses, chickens, Taiwan

## Abstract

A previously unidentified strain of avian hepatitis E virus (aHEV) is now endemic among chickens in Taiwan. Analysis showed that the virus is 81.5%–86.5% similar to other aHEVs. In Taiwan, aHEV infection has been reported in chickens without aHEV exposure, suggesting transmission from asymptomatic cases or repeated introduction through an unknown common source(s).

Avian hepatitis E virus (aHEV) was first isolated from chickens with big liver and spleen disease or hepatitis-splenomegaly syndrome (*1*,*2*). aHEV infection in chickens can cause death and reduce egg production, resulting in economic losses in the poultry industry (*3*). The zoonotic characteristic of aHEV have not been verified with certainty (*4*); however, the virus may have public health implications related to the consumption of contaminated poultry eggs and meat, the use of poultry viscera as a culinary delicacy, and the handling of poultry.

In Taiwan, the prevalence of aHEV in avian livestock has been increasing, but the causative strain has not been known. To increase our knowledge of this growing problem, we determined the seroprevalence of aHEV antibody in chickens in Taiwan and then isolated the infecting virus and sequenced and phylogenetically analyzed its full genome to better determine the origin and evolutionary status of the virus.

## The Study

In 2013, we analyzed serum samples from 1,326 chickens in 61 flocks throughout Taiwan to study the prevalence of aHEV antibodies. In addition, we collected bile samples from 150 chickens among the 4 commercial egg-layer flocks in Pingtung County, Taiwan, to isolate and identify the causative aHEV strain. All chickens appeared to be healthy and ranged in age from 30.1 to 62.8 (mean 43.9) weeks for breeders and from 19.0 to 65.1 (mean 53.1) weeks for layers (Table 1). We tested serum samples for aHEV antibodies by using an ELISA (BioChek, Reeuwijk, the Netherlands) essentially as described by the manufacturer. aHEV antibody seroprevalence was 40.57% (538/1,326) among the chickens and 95.08% (58/61) among the flocks (Table 1).

**Table 1:** Results of avian hepatitis E antibody testing in serum samples from chickens, Taiwan, 2013*

Type of chicken, mean age, wk	No. flocks tested	No. (%) positive		No. chickens tested	No. (%) positive
Breeders, 43.9	41	39 (97.22)		857	294 (34.31)
Layers, 53.1	20	19 (95.00)		469	244 (52.03)
Total, 47.1	61	58 (95.08)		1,326	538 (40.57)

*ELISA was used to test samples for antibody.

We used reverse transcription PCR to isolate the aHEV RNA genome from chicken bile, and 3 sets of degenerative primers were designed to amplify a specific region of the genome. The first and the second sets of degenerative primers were designed on the basis of multiple sequence alignments derived from the helicase gene in open reading frame (ORF) 1 and the capsid gene in ORF2 (*5*), respectively. Primers based on ORF1 were AHEV F-1/SD, 5′-TGTTATYACACCCACCAARACGYTG-3′ for positions 2,524–2,548; Helic R-1, 5′-CCTCRTGGACCGTWATCGACCC-3′ for positions 2,975–2,954; AHEV F-2/SD, 5′-GCCACGGCTRTTACACCYCAYGT-3′ for positions 2,573–2,595; and Helic R-2, 5′-GACCCRGGRTTCGACTGCTT-3′ for positions 2,958–2,939. Primers based on ORF2 were AHEV ORF2/F-1/SD, 5′-TCGCCYGGTAAYACWAATGC-3′ for positions 5,473–5,492; AHEV ORF2/R-1/SD, 5′-GCGTTSCCSACAGGYCGGCC-3′ for positions 5,750–5,731; AHEV ORF2/F-2/SD, 5′-ACWAATGCYAGGGTCACCCG-3′ for positions 5,485–5,504; and AHEV ORF2/R-2/SD, 5′-ATGTACTGRCCRCTSGCCGC-3′ for positions 5,726–5,707.

The third primer set was designed on the basis of 5 multiple alignments of complete or nearly complete aHEV sequences of other aHEV strains (GenBank accession nos. AM943647, GU954430, AM943646, EF206691, and AY535004) for the aligned results near the end of ORF2. The primers included nt5883/F, 5′-GGAYTATGGGAAYCAGCATG-3′ for positions 5,862–5,881; nt6579/R, 5′-ATCACAATAAATTAAACATAGGG-3′ for positions 6,600–6,578; nt6216/F, 5′-TGGGGRCCYCAGGGCGCTG-3′ for positions 6,196–6,214; and nt6498/R 5′-GAGGGGAATGTYYTACTAAG-3′ for positions 6,515–6,496.

Following the primer walking strategy, we designed sequencing primers as detailed in Table 2. We then sequenced the complete genome (6,653 bp) of the aHEV strain isolated from chickens in Taiwan (TWNaHEV; GenBank accession no. KF511797) and determined that it is 1 base pair shorter than that of the prototype aHEV (*6*). The cloned sequence of TWNaHEV RNA is composed of the noncoding region at the 5′ end (1–25 nt); ORF1 (26–4,618 nt), including methytransferase (191–742 nt), helicase (2,429–3,124 nt), and RNA-dependent RNA polymerase (3,167–4,618 nt); ORF3 (4,652–4,915 nt); ORF2 (4,705−6,525 nt); and the noncoding region at the 3′ end (6,525–6,653 nt).

**Table 2:** Primers used in the generation of the complete genome of the Taiwan avian hepatitis E virus strain*

Primer name†	Primer sequence, 5′→3′	Direction
TWN6490–6470	CTAGAAGTCGGCGTGTCTCAG	R
TWN6469–6448	GTGACTGGTCCTCAGGTGCTTG	R
TWN6244–6270	CAGGAGTGGATCTATTTCCTTCAGAAC	F
TWN6243–6262	CCAGGAGTGGATCTATTTCC	F
TWN6215–6241	GATACTTCTATCAGTACAACAACACAC	F
TWN5705–5686	CGCAGCAGCGTGGATGGTAG	R
TWN5506–5526	GTTAAGGTGACTGCTCCGCAC	F
TWN5043–5023	GTCCTGAAGTGGCATGAGCGG	R
TWN4784–4763	CTTCCGGCTGGGAGCGTTTGGG	R
TWN3421–3441	CCATTGTCGCCTGGCTGCACC	F
TWN3227–3247	GACGGGTTATTGGATATACCG	F
TWN2952–2932	GGGTTCGACTGCTTGGCCACC	R
TWN2930–2909	GAGTAAACACAATTTTTTGGCC	R
TWN2569–2588	CGGGGCTGTTGCGATTACGC	F
TWN1657–1635	CATAATGTGCAACGATGGCGGCG	R
TWN1607–1582	CAGCAAGCTCTTTAAGTGTGAGTAGC	R
TWN1570–1546	GAGGTCAATCAAATTCTCAGTGCTG	R
TWN1145–1123	CAGCAATGGCAACAGCCGTCAGC	R
TWN1114–1091	CTCAGGCTGCCAACCCTCATTGGC	R
TWN1048–1026	GTAGGTCAGCAAGCGCGAGCAGC	R
TWN670–646	CTTGTCCGTTGTATTTACGGTATTG	R
TWN607–584	CTCCTCTGGTAAGTGCAACACGAC	R
TWN580–557	CAGTGTCCGCATATTATGGCGGGC	R
HaHEV1–26‡	GCGGCCGCTCTAGCTGCAGCGAATAC	F
HaHEV1410–1433‡	CATCCGTGCGGGTACTAAATCTGC	F
Anchored-oligo (dT)18 primer	NVTTTTTTTTTTTTTTTTTT	NA
Oligo d(T)-anchor primer	GACCACGCGTATCGATGTCGACTTTTTTTTTTTTTTTTV	NA

*The annealing temperature was set at 60°C for each primer combination. R, reverse; F, forward; NA, not applicable. †Numbers following TWN in primer names is the nucleotide position extracted by the respective primers. ‡Primers were designed on the basis of the sequence from Hungary (GenBank accession no. JN997392).

We compared the sequence of strain TWNaHEV with complete or near-complete sequences in GenBank for 7 other aHEVs that had been isolated from chickens; TWNaHEV shared 81.5%–86.5% sequence identity (GenBank accession nos. AM943647 and JN997392, respectively) with the other aHEVs. Phylogenetic analysis using the maximum likelihood method (*7*) with 1,000 bootstraps, based on the sequence variations in nucleotides, indicated high support for a close relationship (98%) between the aHEV genotype IV strains from Hungary and Taiwan (GenBank accession nos. JN997392 and KF511797, respectively) (Figure) (*8*). In addition, the aHEV genotype IV strains are close to the genotype III strains, which are represented by another strain from Hungary and a strain from China (GenBank accession nos. AM943646 and GU954430, respectively), but this relationship is supported by a bootstrap value of only 55%. Moreover, previous studies (*9*–*13*) suggested an association of genetic types of aHEV with geographic regions. In contrast, we found a mixture of strains from Taiwan, China, and Hungary (GenBank accession nos. KF511797; GU954430; and JN997392 and AM943646, respectively) classified into the separate genotype III and IV clades (Figure).

**Figure:**
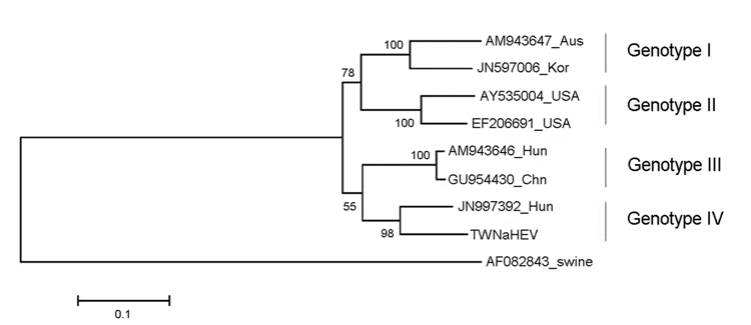
Phylogenetic tree was aligned by using the maximum likelihood method (1,000 bootstraps) for 8 complete or nearly complete avian hepatitis E (aHEV) sequences and a swine HEV outgroup. GenBank accession numbers, country abbreviations, and avian genotype are indicated. Scale bar indicates nucleotide substitutions per site.

## Conclusions

Several cases of aHEV in chickens without aHEV exposure have been reported in Taiwan. No apparent full-scale outbreaks of acute or chronic aHEV disease have occurred, yet the estimated high seroprevalence of aHEV antibodies among chickens in Taiwan indicates that the disease is now endemic. This finding suggests the possibility of aHEV transmission from asymptomatic cases or repeated introduction through an unknown common source(s). Studies on public health issues related to aHEV; the geographic prevalence and genetic diversity of aHEV; and cross-species infection with aHEV are lacking, and studies on the zoonotic properties of aHEV are incomplete but underway. Knowledge of the diffusion pattern of aHEV around Taiwan is also lacking, although it is known that horizontal, but not vertical, transmission of aHEV is possible (*12*,*14*). Given these facts, hepatitis surveillance is essential in Taiwan.
